# National Medical Convention

**Published:** 1845-10

**Authors:** 


### MEDICAL INTELLIGENCE.

*National Medical Convention.*—In our last number, in our Notice of the Transactions of the Medical Society of the State of New York, we called the attention of our readers to the resolutions of that body, recommending a *National Medical Convention*, to be held in the city of New York on the first Tuesday in May, 1846. Since then, we have received the circular of the committee appointed to carry those resolutions into effect, accompanied by a letter from Dr. N. S. Davis, one of the committee, inviting, through us, the medical colleges of the State of Illinois, and the medical societies of this State, (if any exist,) to participate, by their delegates, in the convention. The objects of the Medical Society of the State of New York, in calling the convention, as stated in the letter of Dr. Davis, "were, to freely discuss the great and paramount interests of the whole profession of this country; to make the members of the profession in each State better acquainted with the men and institutions of every other State; to excite and diffuse a spirit of scientific and professional investigation; and, if possible, devise some concerted plan for *elevating the practical standard of medical education* throughout the whole country." Are not these objects of sufficient importance to enlist the interest and exertions of the medical profession, to carry into effect the only plan which can secure their attainment? We ask for the subject the earnest attention of all our readers, and their adoption, individually and collectively, of such measures as will forward the plan proposed. It would give us pleasure to hear from all who are interested in the movement, and to join with them in the effort to secure for our State a representation in the National Convention. Our pages will be open to communication upon the subject, suggesting the mode to be adopted for that purpose. We earnestly hope the subject will meet the attention it merits. Ed.

